# The Salivary Scavenger and Agglutinin (SALSA) in Healthy and Complicated Pregnancy

**DOI:** 10.1371/journal.pone.0147867

**Published:** 2016-02-01

**Authors:** Martin Parnov Reichhardt, Hanna Jarva, Anna Inkeri Lokki, Hannele Laivuori, FINNPEC study group, Piia Vuorela, Vuokko Loimaranta, Andreas Glasner, Monika Siwetz, Berthold Huppertz, Seppo Meri

**Affiliations:** 1 Immunobiology Research Program, Research Programs Unit, and Department of Bacteriology & Immunology, Medical Faculty, University of Helsinki, Helsinki, Finland; 2 Helsinki University Hospital Laboratory (HUSLAB), Helsinki, Finland; 3 Medical Genetics, University of Helsinki and Helsinki University Hospital, Helsinki, Finland; 4 Obstetrics and Gynecology, University of Helsinki and Helsinki University Hospital, Helsinki, Finland; 5 Institute for Molecular Medicine Finland, University of Helsinki, Helsinki, Finland; 6 Obstetrics and Gynecology, Porvoo Hospital, Porvoo, Finland; 7 Department of Medical Biochemistry and Genetics, University of Turku, Turku, Finland; 8 Femina-Med Center, Graz, Austria; 9 Institute of Cell Biology, Histology and Embryology, Medical University of Graz, Graz, Austria; 10 Biobank Graz, Organizational Unit of Research Infrastructure, Medical University of Graz, Graz, Austria; Otto-von-Guericke University Magdeburg, Medical Faculty, GERMANY

## Abstract

Pre-eclampsia is a leading cause of maternal and perinatal morbidity and mortality worldwide. The etiology is not clear, but an immune attack towards components of placenta or fetus has been indicated. This involves activation of the complement system in the placenta. We have previously described the presence of the complement-regulating protein salivary scavenger and agglutinin (SALSA) in amniotic fluid. In this study we investigated the potential role of SALSA in pregnancy by analyzing its presence in amniotic fluid and placental tissue during healthy and complicated pregnancies. SALSA levels in amniotic fluid increased during pregnancy. Before 20 weeks of gestation the levels were slightly higher in patients who later developed pre-eclampsia than in gestation age-matched controls. In the placenta of pre-eclamptic patients syncytial damage is often followed by the formation of fibrinoid structures. SALSA was found clustered into these fibrinoid structures in partial co-localization with complement C1q and fibronectin. *In vitro* analysis showed direct protein binding of SALSA to fibronectin. SALSA binds also to fibrin/fibrinogen but did not interfere with the blood clotting process *in vitro*. Thus, in addition to antimicrobial defense and epithelial differentiation, the data presented here suggest that SALSA, together with fibronectin and C1q, may be involved in the containment of injured placental structures into fibrinoids.

## Introduction

Pregnancy introduces a temporary condition, where two humans co-exist, one inside the other. During this period, fetus and placenta may express and secrete paternal antigens. Thus, this symbiosis challenges the maternal immune defense system to develop tolerance to these new antigens. Pre-eclampsia (PE) is a common pregnancy-related complication affecting 3% of all pregnancies. It is the leading cause of maternal and perinatal morbidity and mortality worldwide [1,2]. The etiology of PE is currently unclear. Many observations point towards the possibility that an immune incompatibility between the feto-placental unit and the mother could be involved. Similar factors could also contribute to other pregnancy complications such as intrauterine growth restriction (IUGR) and premature birth.

Specific characteristics of PE include intravascular inflammation with elevated levels of pro-inflammatory cytokines in the maternal circulation, and endothelial cell activation and/or dysfunction [3–6]. In concert with these findings, PE is often accompanied by platelet and thrombin activation, leading to thrombocytopenia and a risk for intravascular coagulation [7]. A prominent feature of PE is the activation of the coagulation cascade, e.g. through activation of thrombin [8,9]. As a sign of both injury and activation of the coagulation cascade lesions known as fibrinoids can be detected in the placenta and occasionally also in various organs of the maternal body [10]. Fibrinoids are generally divided into two categories, matrix-type and fibrin-type fibrinoids [11]. Matrix-type fibrinoid is generated by invading extravillous trophoblast cells. Fibrin-type fibrinoid resembles blood clots as seen in other tissues, and may be deposited where blood flow is impaired or where the syncytiotrophoblast layer is injured or interrupted [11]. Excessive syncytial damage with fibrinoid formation is a common feature of complicated pregnancies. However, fibrinoids may also be observed in healthy placentas [11].

The complement system is an essential part of the innate immune defense system with well-established functions in both the protection against invading pathogens as well as in waste disposal of e.g. apoptotic and injured cell material. The complement system is composed of three pathways initiated by binding of pattern recognition molecules, such as C1q (classical pathway), mannose binding lectin (MBL) and ficolins (lectin pathway). The third (alternative) pathway recognizes a wide variety of nonself structures and functions as an amplification loop for complement activation initiated via other pathways [12,13]. During pregnancy there is a general suppression of the immune system to protect the fetus. Under healthy conditions, the immune system, including the complement system, is still able to clear injured and necrotic tissues. However, in some cases malfunction of this process may occur and the deposition of this material into fibrinoid clusters at the surface of placental villi and other sites could cause inflammation. In time this may lead to endothelial-vascular disorders and a proinflammatory state in the maternal circulation [14].

The salivary scavenger and agglutinin (SALSA), also known as gp340, salivary agglutinin (SAG) and deleted in malignant brain tumor 1 (DMBT1) is a glycoprotein of 340 kDa (Genbank accession no. BAA78577.1) [15–17]. SALSA belongs to an ancient family of scavenger receptors, the scavenger receptor cysteine-rich (SRCR) protein family, identified by multiple repeats of the highly conserved SRCR domains [18]. SALSA is produced by mucosal tissues throughout the body. The protein is found to be associated with the epithelial layers in the lung, mouth, trachea, gastrointestinal tract, vagina and skin [18–22]. However, studies have found that SALSA is also expressed sporadically in the endothelial cells of cardiac capillaries and in capillaries of skeletal muscle, at least in mice [23,24]. Several secreted forms of SALSA have been found in body fluids lining the mucosal surfaces such as saliva, lacrimal fluid, pancreatic juice and as well in bronchoalveolar lavage [15,16,25,26]. We recently observed that SALSA is very abundant in the infant intestine and amniotic fluid (AF) [27]. However, so far it has not been described in blood. Recent advances in the understanding of the physiological function of SALSA point towards a role in both epithelial cell differentiation and innate immunity [28].

The complement components C1q and MBL bind SALSA and through these interactions SALSA has been shown to regulate complement activation in solution and on surfaces [29–31]. The pathogenesis of pregnancy complications, in particular PE, has been associated with a regulatory disturbance in immune responses and specifically in complement activation. We therefore decided to further localize the SALSA protein in healthy and complicated pregnancies. Our approach aimed to elucidate the role of SALSA in pregnancy by measuring its levels in AF samples from women with normal and complicated pregnancies, localizing the SALSA protein in placenta and determining its related molecular targets and interaction partners in placental lesions.

## Materials and Methods

### Amniotic fluid samples

AF was collected at the Department of Obstetrics and Gynecology, Helsinki University Hospital, Helsinki, Finland. Samples from second trimester (17.2 ± 2.5 gestational weeks) were originally collected for screening of fetal chromosomes or assessment of alpha-fetoprotein. All karyotypes and alpha-fetoprotein levels were normal. Samples from second trimester are referred to as early pregnancy. AF samples from the third trimester (term pregnancy) were collected by amniocentesis for assessment of fetal lung maturity or during caesarean section from women with PE (gestational week 34 ± 2.3, n = 6), IUGR (gestational week 34.2 ± 3.6, n = 15), diabetes mellitus type 1 (DM, gestational week 36.9 ± 1.2, n = 19) or gestational diabetes mellitus (GDM, gestational week 36.5 ± 2.5, n = 17). Control AF samples from women with uncomplicated pregnancies (gestational week 39.4 ± 1.0, n = 14) were collected by needle aspiration during an elective caesarean section made because of breech presentation or fetopelvic disproportion. After collection the samples were immediately frozen and stored at– 20°C. All amniotic fluid samples were collected after informed written consent, and use of the samples was approved by the Medical University Graz, ethical commission (26–132 ex 13/) and the ethical committee of the Hospital District of Helsinki and Uusimaa (14149/E0/07).

### Placental samples

Placental samples were obtained from different cohorts. The following paraffin-embedded tissue samples were obtained from Medical University of Graz, Austria. First trimester placentas were obtained after elective termination of pregnancy for social reasons (gestational weeks 8–11, n = 3). Early onset PE-placentas (gestational weeks 29–34, n = 10) and age-matched controls (gestational weeks 29–34, n = 7) were obtained after caesarean section. Healthy term placentas (gestational weeks 36–40, n = 5) were obtained after vaginal delivery. The use of placental samples was approved by the Medical University Graz, ethical committee (26–132 ex 13/14). Frozen placental tissue from PE (gestational weeks 35.3 ± 3.7, n = 13) and healthy (gestational weeks 39.7 ± 1.5, n = 10) pregnancies were obtained from the Finnish Genetics of Pre-eclampsia Consortium (FINNPEC) cohort including individuals delivering at the Helsinki University Central Hospital, Finland. PE and healthy women delivered both by caesarean section and vaginal delivery. FINNPEC study protocol was approved by the coordinating ethical committee of the Hospital District of Helsinki and Uusimaa (14149/E0/07). Detailed description of the cohort has been reported previously [14]. Individuals with multiple pregnancies, maternal age <18 years or known autoimmune diseases were excluded. All subjects gave written informed consents.

### Quantification of SALSA in AF by ELISA

To quantify the levels of SALSA, samples were diluted and coated directly onto Maxisorp plates (Nunc, Denmark). SALSA purified from saliva was used as a protein concentration standard. After coating, the plates were blocked with 5% nonfat milk in TBS/1 mM Ca^2+^. The plates were washed with TBS/Ca^2+^ and 0.05% Tween-20 (TBS/Ca^2+^/Tween). SALSA levels were detected using monoclonal anti-SALSA (Hyb 213–06, Bioporto, Denmark) diluted to 0.05 μg/ml and HRP-conjugated rabbit anti-mouse antibodies (Jackson ImmunoResearch Laboratories, West Grove, PA) diluted 1:10 000 in TBS/Ca. OPD tablets (Dako, Denmark) were used for development and the color reaction was measured with an iEMS Reader MF (Labsystems, Espoo, Finland) at an OD of 492 nm. For each sample a dilution series was made to ensure that the readings were within the linear range. Measurements were based on a minimum of three separate assays.

### Immunohistochemistry

Sections of paraffin-embedded tissues (5 μm) were dried O/N at 45°C and subjected to standard de-paraffination followed by antigen retrieval treatment in a pressure cooker at pH 9, 120°C and 15 psi pressure. Sections were washed briefly in TBS/ 0.05% Tween and blocked in UltraVision Hydrogen Peroxide Block (Thermo Fisher Scientific, Waltham, MA, USA) followed by UltraVision Protein Block. The staining protocol followed manufacturer recommendations of the kit UltraVision LP Large Volume Detection System (HRP Polymer Ready-To-Use, Thermo Fisher Scientific) with few specifications. Briefly, sections were washed four times in TBS/Tween. For incubation (30 min, 37°C) anti-SALSA (Hyb 213–06) was diluted to a final concentration of 10 μg/ml in antibody diluent (Dako) followed by incubation with primary antibody enhancer, HRP Polymer, 3-Amino-9-Ethylcarbazole (All ThermoFisher Scientific) and finally counterstain by Mayer's hemalum followed by mounting by Aquatex (Merck, Darmstadt, Germany).

### Fluorescence microscopy and immunohistochemistry

Sections of paraffin-embedded tissues were prepared as above. Frozen sections (5 μm) of healthy and PE placentas were prepared by cryosectioning and stored at -70°C. The sections were then thawed and rinsed briefly in TBS/Ca^2+^ before use. The sections were blocked in TBS/Ca^2+^ with 1% bovine serum albumin (BSA) for 30 min at 37°C in a humid chamber. Antibodies were diluted in TBS/Ca^2+^/BSA and incubated 30 min at 37°C in a humid chamber. Between incubations the sections were washed 3 × 5 min in TBS/Ca^2+^/BSA. Anti-SALSA was diluted to 10 μg/ml. For co-localization studies PBS/0.05% Tween was used for washes while polyclonal rabbit anti-cellular fibronectin (ab299, Abcam, Cambridge, United Kingdom) and rabbit anti-C1q (Dako) antibodies were used 1:1000 in DAKO antibody diluent and Alexa 488 labeled goat anti-rabbit and Alexa 546 labeled goat anti-mouse antibodies (both Invitrogen, Oregon, USA) were used at 1:300 dilutions in PBS. Classical and lectin pathway activation was investigated by staining with polyclonal rabbit anti-C4c (Dako) and Alexa 488 labeled goat anti-rabbit (Invitrogen) diluted 1:400 and 1:300 in PBS, respectively. Nuclei were stained with DAPI (Invitrogen, Molecular Probes, Eugene, Oregon, USA) diluted 1:2000 in PBS. *Ex vivo* SALSA binding was tested using AF as a biological source of SALSA. An overlay was performed with un-diluted AF after initial blocking on frozen sections. Following this the antibodies were added as described. After final incubation and wash, excess liquid was removed and a mounting liquid was added. The sections were then immediately analyzed by fluorescence microscopy in Finland: Olympus DP Manager (ver. 2.2.1.195) and Olympus DP Controller (version 2.2.1.227) image capture softwares with Olympus BX51 fluorescence microscope camera, and in Austria: VIS Visiopharm Integrator System (version: 4.5.1.324) software for multichannel image acquisition and Leica microscope with Olympus DP72 camera.

### Effect of SALSA on coagulation

Basic coagulation assays such as Thrombin Time and Activated Prothrombin Time measurements were performed as described [32]. In short, Thrombin Time measurements were performed by adding 100 μl BC Thrombin reagent (Siemens, Germany) to 40 μl citrated plasma. Citrated plasma was taken into 3.2% sodium citrate 9NC anti-coagulation tubes (Greiner Bio-One, Kremsmünster, Austria) and separated by centrifugation at 2500×g. Coagulation time was measured using a coagulometer. Activated Prothrombin Time measurements were performed by mixing 50 μl Dade Actin FSL reagent (Siemens) with 50 μl citrated plasma. After a 3 min incubation, 50 μl 0.025 mol/l CaCl_2_ was added, and the coagulation time was measured using a coagulometer. For both assays, SALSA was mixed with plasma in the fluid phase prior to the start of coagulation at final concentrations of 0 μg/ml, 1 μg/ml, 3 μg/ml and 5 μg/ml.

The effect of surface coated SALSA on coagulation was tested in an assay modified from the protocol published by Rose and Babensee [33]. SALSA was coated at 1 μg/ml on a Maxisorp plate. 100 μl citrated plasma was heated to 37°C and added to wells coated with SALSA or wells without SALSA. 100 μl BC Thrombin reagent was heated to 37°C and added to initiate coagulation. OD405 measurements were made every 20s for 30 min using a FLUOstar optima reader (BMG Labtech, Offenburg, Germany). The increase in absorbance corresponds to the development of the clot.

### SALSA binding to fibronectin

Binding of SALSA to human plasma fibronectin was tested in an ELISA setup. Maxisorp plates were coated with 1, 5, or 10 μg/ml of human plasma fibronectin (Chemicon, CA, USA) in Na_2_CO_3_-buffer, pH 9.6 O/N at 4°C. Plates were washed 3 times with 0.5 mM NaCl, 20 mM Tris, 0.05% Tween 20, pH 7.4 (TTSB) and blocked with 3% BSA in TTSB. After 2 hours at RT plates were washed and SALSA (1 μg/ml in TTSB + 0.1% BSA with or without 1mM Ca^2+^) was added and incubated for 60 min, RT. Binding was detected using monoclonal anti-SALSA antibody Hyb 213–06 (0.4 μg/ml in TTSB/BSA/Ca^2+^) and HRP-conjugated rabbit anti-mouse antibodies (1:10 000 in TBS/Ca^2+^). OPD tablets were used for development and the color reaction was measured. Each experiment was performed three times.

## Results

### Amniotic fluid SALSA levels in normal and complicated pregnancies

**Fig 1:**
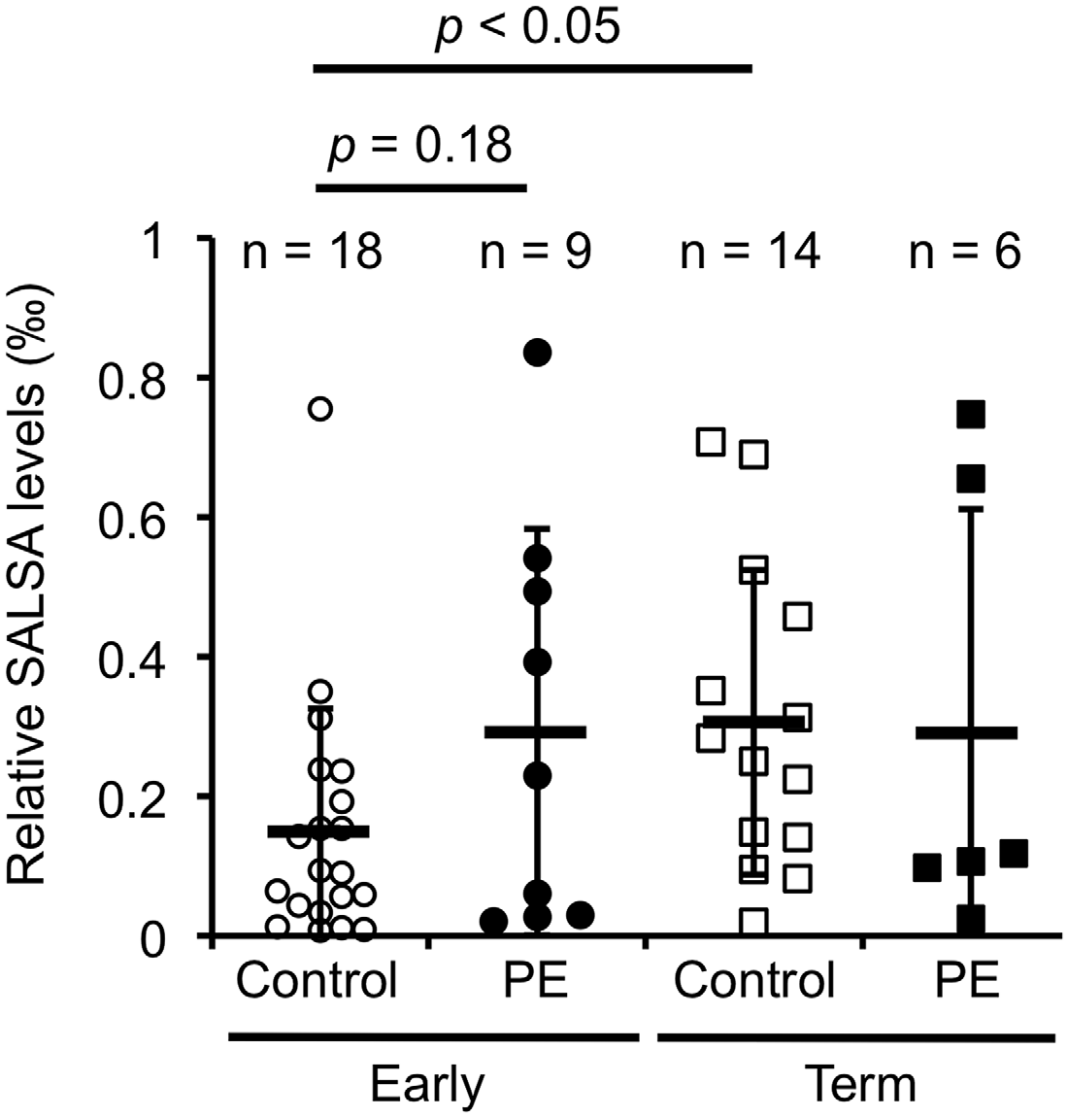
SALSA levels in the amniotic fluid (AF) from healthy and complicated pregnancies. SALSA was measured by ELISA. SALSA levels are shown relative to the total protein amount. Comparing samples taken before 20^th^ week of gestation from women who at term were either pre-eclamptic (PE, n = 9) or healthy (n = 18), we found a trend of higher SALSA levels in PE pregnancies. However the difference was not significant (0.32 ‰ ± 0.3 vs. 0.17 ‰ ± 0.18, Students t-test: *p* = 0.18). When comparing SALSA levels in AF from healthy term (n = 14) and PE (n = 6) pregnancies no significant differences between the groups were found (Student’s t-test, *p* > 0.1 for all). SALSA levels in AF from healthy pregnancies before 20^th^ week of gestation (n = 18) and at delivery (n = 14) were shown to double from 0.17 ‰ ± 0.18 to 0.31 ‰ ± 0.21 of the total protein amount (Student’s t-test: *p* < 0.05).

In our previous study we observed SALSA in AF from healthy pregnancies by immunoblotting. In an ELISA analysis the concentrations of SALSA ranged between 0–11.5 μg/ml (mean: 2.1 ± 3.7 μg/ml) [27]. To identify a possible role for SALSA in the pathogenesis of pregnancy complications, SALSA levels were measured in AF from patients diagnosed with IUGR, PE, GDM or DM and compared to healthy controls. Included in the analysis were additional samples taken at an early stage (before 20 weeks of gestation) from patients who later developed PE compared to age-matched controls. Levels of SALSA were measured by ELISA. The overall protein level of the AF is known to vary during the course of pregnancy. Thus, to make the samples comparable, SALSA concentrations were related to total protein levels of the AF samples (Fig 1).

The pathological process of PE assumingly starts much earlier than the manifestation of symptoms [34]. Comparison of the SALSA levels at an early stage and at term could provide information on the expression of SALSA during the maturation of the fetus and possible role in PE. First, samples were collected from healthy pregnancies at an early stage (before gestational week 20, n = 18) and at term (n = 14) and SALSA levels were measured by ELISA (Fig 1). The concentration of SALSA in AF increased during the maturation of the fetus. The actual measured concentrations increased from 0.7 (± 0.5) μg/ml in early pregnancy to 2.1 (± 1.6) μg/ml at term (*p*<0.0006). The corresponding total protein levels in AF increased from 5.3 mg/ml to 6.8 mg/ml (*p*<0.01). Thus, the relative SALSA level doubled from 0.15 ‰ to 0.3 ‰ of the total protein amount (*p*<0.05). This was corroborated by a positive Spearman correlation test between the relative SALSA levels and the gestational week at sampling with a correlation coefficient of 0.348 (*p*<0.05).

As shown in Fig 1 we observed variations in SALSA levels in early pregnancy in both patients who later developed PE and healthy controls. The concentrations of SALSA were 0.7 ± 0.5 μg/ml (n = 18) for the control group and 1.4 ± 1.4 μg/ml (n = 9) for the PE group (*p* = 0.09). When the values were related to the total protein levels the values were 0.17 ± 1.8 ‰ for controls and 0.29 ± 0.29 ‰ for PE. These differences were not significant, but indicated a trend towards increased levels of SALSA at an early time point of patients who later developed PE. At term no differences were found between SALSA levels in healthy controls and PE patients. SALSA levels were also investigated for patients with IUGR, DM and GDM, however no differences were found.

### Deposition of SALSA in placenta

**Fig 2:**
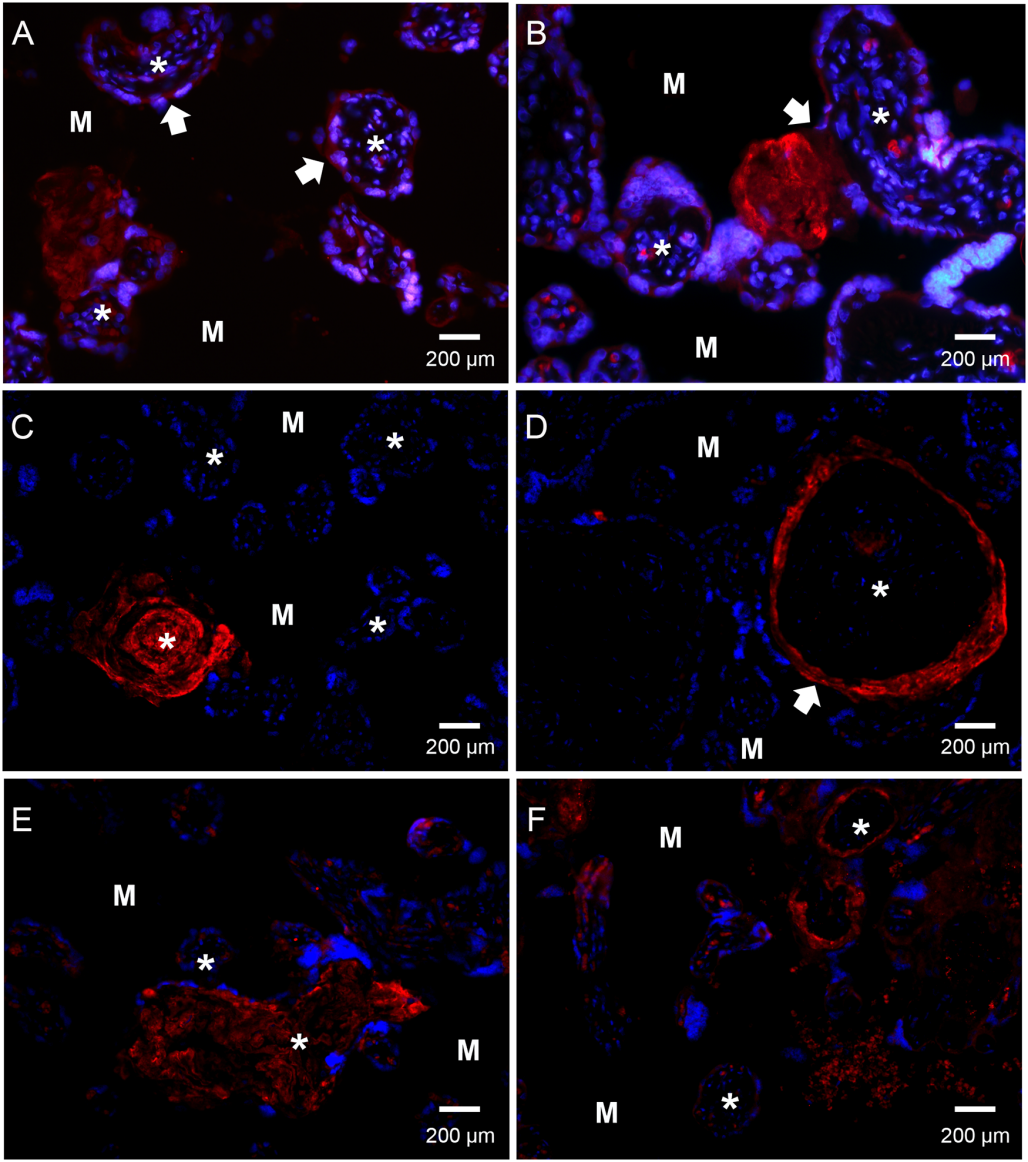
Immunofluorescence microscopy detection of SALSA localization in human placenta. Paraffin-embedded tissue from healthy (panels A-D) and pre-eclamptic (PE) (panels E-F) placentas. Sections were stained with an anti-SALSA antibody (Hyb 213–06) and Alexa 546-conjugated goat anti-mouse IgG. Red: SALSA, blue: DAPI. * denotes the center of the villus structures. M denotes the intervillous space/maternal tissue. Panels A to F show a widespread but focal staining of SALSA in the human placenta. Based on the morphology and localization of the placental structures SALSA appears to be present in fibrinoid formations, especially in relation to a disruption of the syncytiotrophoblast layer. (A) shows the expression of SALSA found intracellularly in the syncytiotrophoblast layer of some, but not all, villi (white arrowhead). SALSA was observed abundantly in fibrinoid structures at the edge of the villi. A breach of the syncytiotrophoblast layer accompanied by formation of SALSA-positive fibrinoid in the intervillous space can be observed (white arrowhead, panel B). (C) shows SALSA in a necrotic villus with fibrinoid formation. (D) shows a villus with disrupted syncytiotrophoblast layer (white arrowhead). Here the disruption may have led to the influx of maternal blood, and SALSA-positive fibrinoid can be observed separating the syncytiotrophoblast layer from the underlying basement membrane. The fibrinoid may deposit all the way around the exposed villus structure, thus forming a ring structure. Syncytial damage and fibrinoid formation is observed more frequently in PE. (E) and (F) show examples of a necrotic villus and a ring formation in PE placentas. 200× magnifications.

AF is a unique body fluid, since it is only present during pregnancy. The same is true for the placenta and the surrounding tissue. Complement component deposits have been found in the placenta during pregnancy. In addition, an increased deposition was seen in complicated pregnancies, such as PE [14]. To analyze the possible role of SALSA in pregnancy we investigated the presence and deposition of SALSA in the placenta (Fig 2).

In Fig 2 both healthy (panels A-D) and pre-eclamptic (panels E and F) placentas were investigated. We observed focal positive staining of SALSA in most placental tissue sections. More detailed investigation of SALSA in the placenta revealed that SALSA is present in fibrinoid formations at various locations in the placenta. Furthermore, a weaker but positive staining of SALSA was also seen intracellularly in the syncytiotrophoblasts. The syncytiotrophoblast layer of some villi stained more strongly than others, while the cytotrophoblasts were negative for SALSA. The most abundant staining was observed in fibrinoids at the edges of the villous structures. Whenever the syncytiotrophoblast layer was damaged or breached SALSA-positive fibrinoids were observed (Fig 2, panels A and B). In placentas of both healthy and PE pregnancies necrotic villous structures were observed. Eventually these had become covered by SALSA-positive fibrinoids, leaving ghost-like formations of the original villi (Fig 2 panels C and E). Disruption of the syncytium may lead to an influx of maternal blood into the fetal side of the placenta. This maternal blood flow may separate the damaged syncytiotrophoblast layer from its basement membrane and fibrinoid may be deposited in between the two layers. These ring-shaped structures were also positive for SALSA (Fig 2 panels D and F). The SALSA positive structures were similar in healthy and PE placentas. However, disruption of the syncytiotrophoblast layer and the presence of fibrinoids is generally observed more frequently in PE placentas [14]. No correlation was found between expression of SALSA and mode of delivery. To investigate the possible origin of AF SALSA, we performed stainings of amniotic membranes. However, only a weak signal was observed (data not shown).

**Fig 3:**
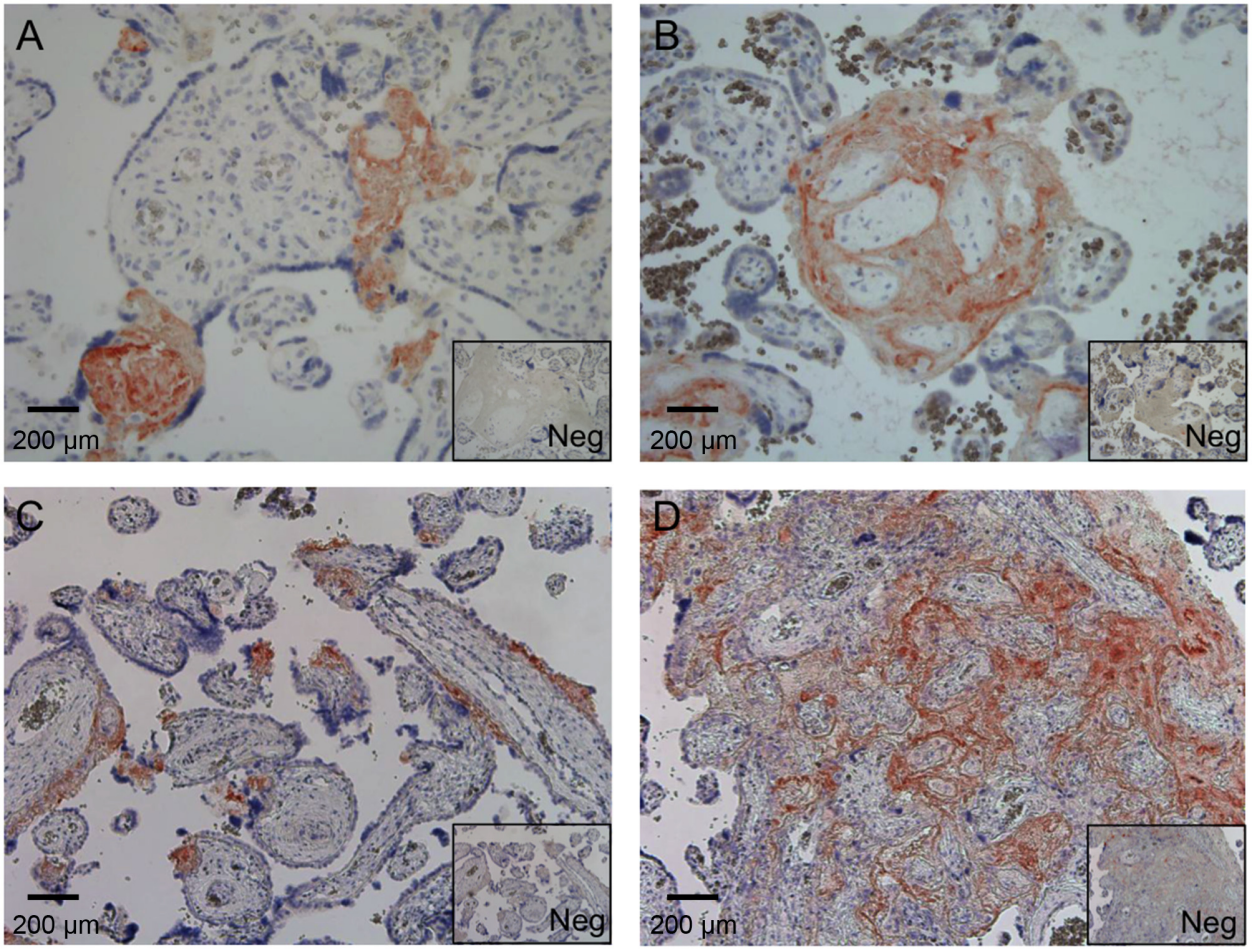
Immunohistochemistry analysis of SALSA in placenta. Paraffin-embedded placental tissue from healthy and pre-eclamptic (PE) pregnancies were stained for SALSA. Both healthy (A) and (B) and PE (C) and (D) sections are displayed with corresponding negative IgG-controls (Neg). Based on the morphology and location of the positively staining structures, SALSA primarily stains fibrin-type fibrinoids. (A) and (D) show staining of specific focal fibrinoid formations in apparently necrotic villous structures. Panel B shows an example of fibrinoid “gluing” of villi. (D) shows a larger necrotic area with massive fibrinoid formation. 200× magnifications.

To verify the observations made by immunofluorescence microscopy and to relate SALSA staining more precisely to tissue structures, immunoperoxidase staining was performed on paraffin-embedded placental sections from healthy and PE placentas (Fig 3). This analysis verified that the SALSA protein is present in fibrinoid structures in both healthy (panels A and B) and PE (panels C and D) placentas. According to current terminology [11] fibrinoids are divided into matrix-type fibrinoids, which are secretion products of extravillous trophoblasts, and fibrin-type fibrinoids, which are coagulation products of maternal blood with large amounts of fibrin. SALSA was found to be present primarily in fibrin-type fibrinoids. The staining was specifically observed within individual necrotic villous structures (Fig 3 panels A and C) and in larger necrotic areas with massive fibrin formation (Fig 3 panels B and D). In addition, SALSA was also seen in fibrin-type fibrinoid formations at the interfaces between placenta and endometrium.

### Expression of SALSA in early pregnancy

**Fig 4:**
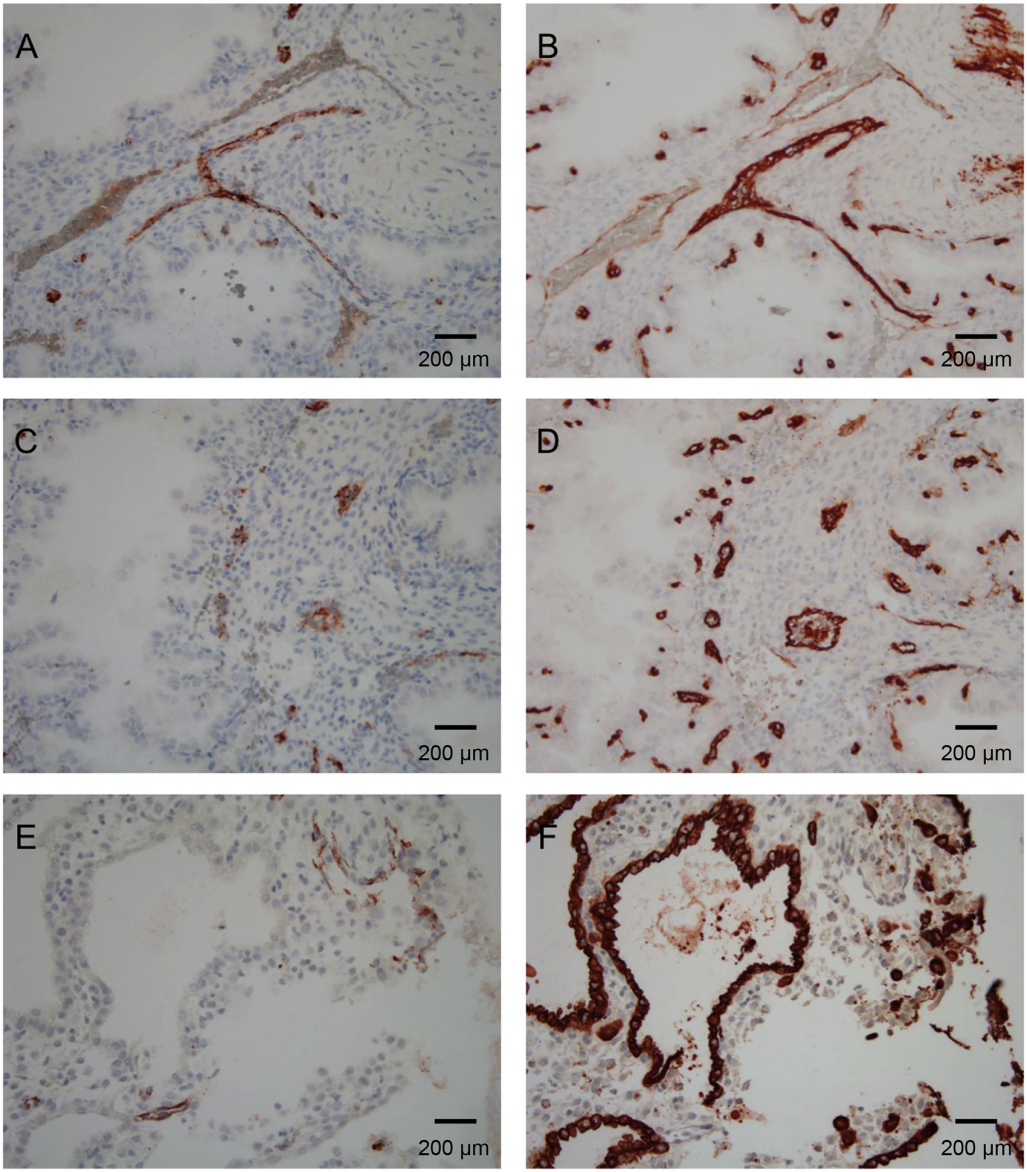
Immunohistochemical analysis of SALSA in paraffin-embedded healthy first trimester decidua. SALSA staining (A), (C) and (E) was compared to the endothelial marker CD34 (B) and (D) and the epithelial marker cytokeratin 7 to identify uterine glands and extravillous trophoblasts (F). In the 1^st^ trimester decidua, SALSA was still found abundantly. However less fibrinoid structures were observed (A) and (C). Instead, SALSA was found to co-localize with CD34 (B) and (D). The SALSA staining pattern of the endothelium is scattered, and found in both small capillaries and larger vessels. Thus, it may be related to the activation state of the endothelium. SALSA was not found to co-localize with cytokeratin 7, and is thus not likely to be produced in the uterine glands (E) and (F). 200× magnifications.

As shown in Fig 1, SALSA levels in the AF seem to increase during the course of pregnancy. To get an insight into the distribution and possible role of SALSA during the development of the placenta, sections from first trimester placentas were also included in the immunohistochemical staining (Fig 4). Fibrinoids were found to be almost absent in the first trimester placenta. We found no staining of SALSA in the early villi. However, positive staining for SALSA was still observed in the decidua. SALSA was present in the endothelia of maternal blood vessels. By comparison to the endothelial marker CD34 we found that a large part of the smaller capillaries and a portion of the larger vessels were positive for SALSA (Fig 4A–4D). The pattern of SALSA staining in the endothelium was somewhat irregular. This could suggest that the presence of SALSA is related to the activation status (or damage) of endothelial cells. By comparison to the marker cytokeratin 7, we could show that SALSA is not present in the extravillous trophoblasts or uterine glands (Fig 4E and 4F). The uterine glands are thus not the source of SALSA in the AF of early pregnancy.

### Binding targets for SALSA in the placenta

**Fig 5:**
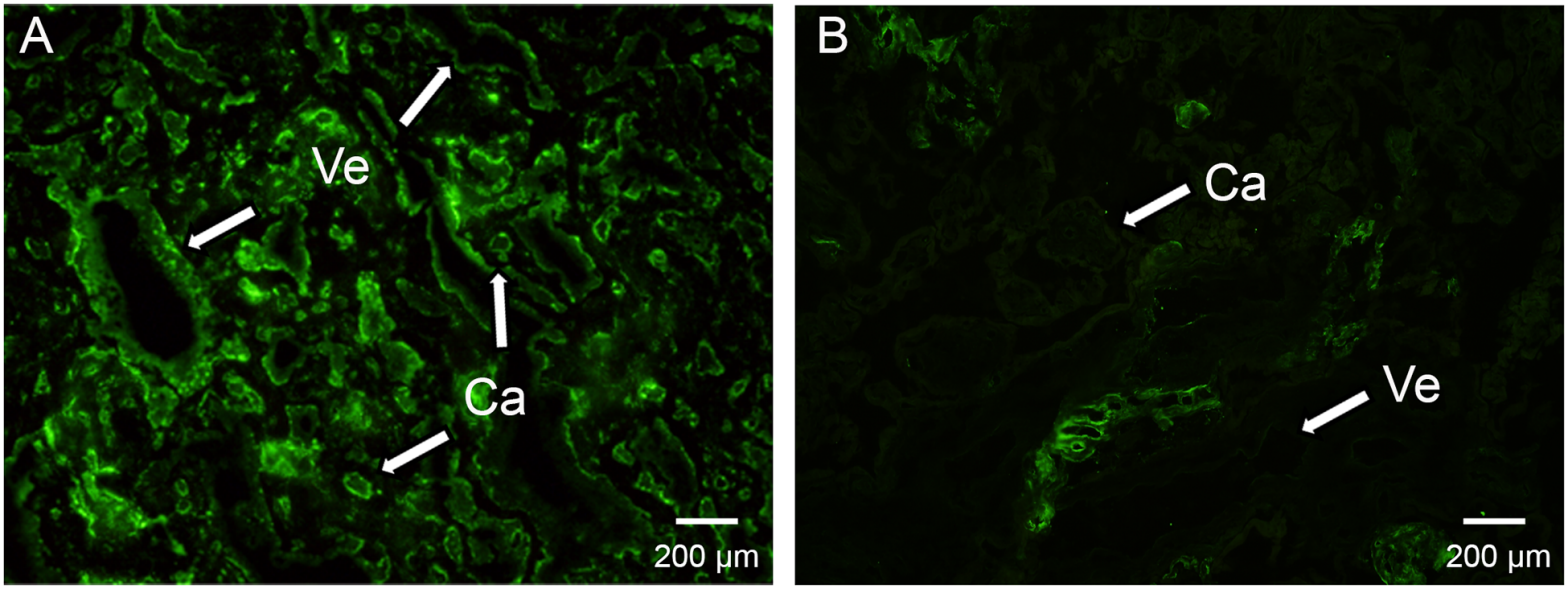
Targets for SALSA in human placenta. To determine further binding sites for SALSA in the placenta, SALSA containing AF was added as an overlay to frozen placental sections prior to detection of SALSA by fluorescence immunohistochemistry. The addition of the SALSA containing overlay (A) revealed a strong binding of SALSA to numerous additional structures in the placenta which were not observed without the overlay (B). SALSA binds to the endothelium of most capillaries (Ca) and larger vessels (Ve) as well as to most of the syncytiotrophoblast layer (A). Without overlay SALSA staining is much more sporadic or lacking in these structures (B). The strong binding after *ex vivo* addition of SALSA suggests that the focal staining observed in the absence of overlay (Fig 2) is a result of limited availability of SALSA rather than the lack of interacting tissue components. 400× magnifications.

So far, the results showed that SALSA was deposited in fibrin-type fibrinoids and necrotic villi at term and irregularly on placental endothelial cells in the first trimester. To identify potential targets for SALSA under *ex vivo* conditions, we performed an overlay of frozen placental sections with SALSA. Undiluted AF was used in the overlay as a source of biologically active SALSA protein (Fig 5). After addition of SALSA containing AF to the frozen placental sections, an increase in positive staining was observed. Binding of SALSA was seen to the syncytial basement membranes and the placental endothelium of most capillaries and also of larger vessels. This indicated that targets for SALSA are present in the endothelium either directly on the surface of the endothelial cells or in the extracellular matrix (ECM). The more limited focal staining observed without the overlay suggested that not only exposure of specific targets, e.g. because of tissue injury, but also the availability of SALSA affects the amount of its deposition in the tissue.

### Effect of SALSA on blood clotting

**Fig 6:**
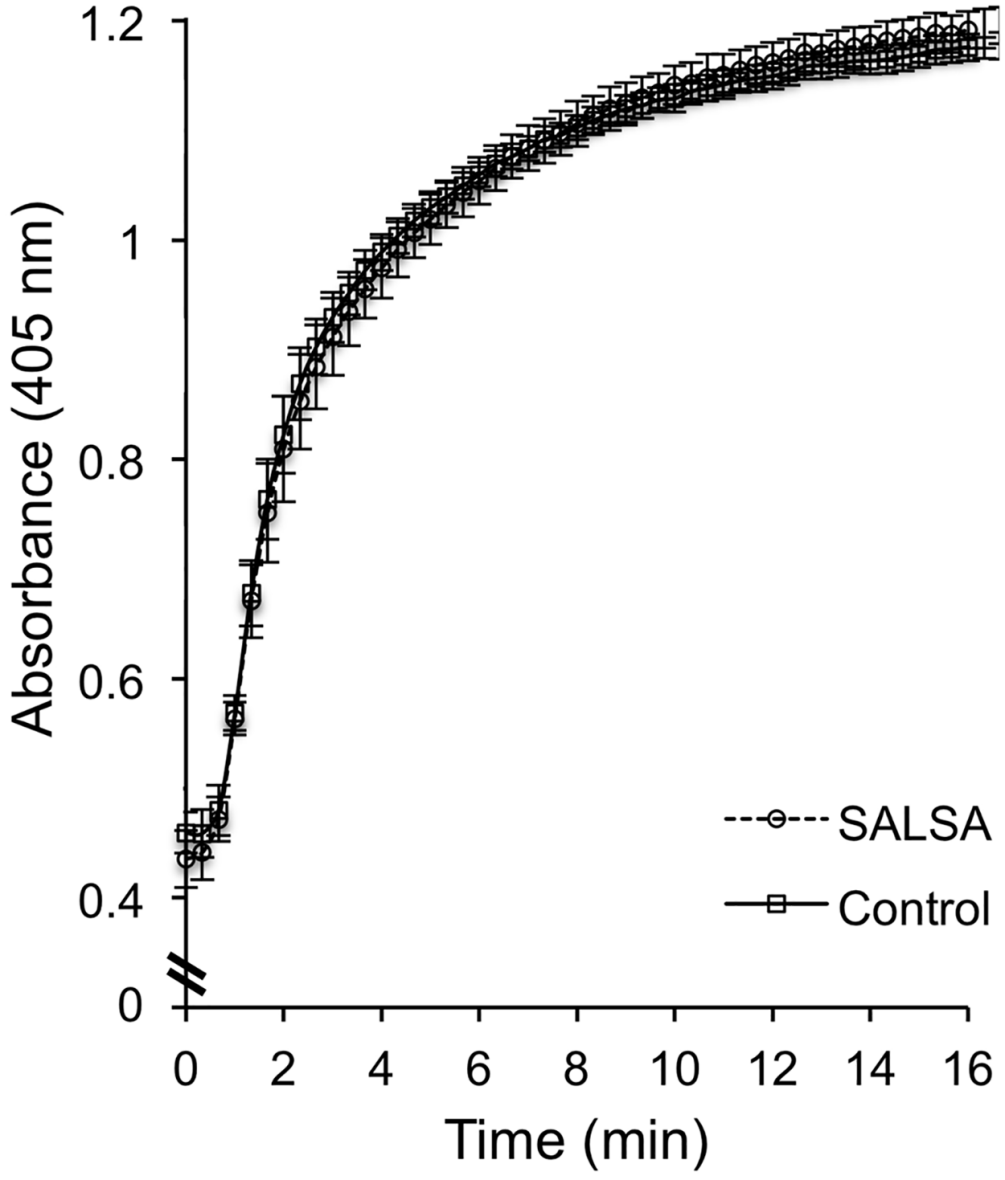
Analysis of the effect of SALSA on coagulation. A pool of citrated plasma was added to SALSA-coated wells (1–5 μg/ml) and coagulation was initiated by adding BC Thrombin reagent at time point 0. The coagulation was followed by absorbance at 405 nm and compared to coagulation in wells without SALSA coating. We did not observe an effect of SASLA on coagulation. Displayed are averages ± SD’s from 5 different wells. A similar result was obtained when soluble SALSA (1–5 μg/ml) was added to the plasma prior to the coagulation test (data not shown).

The overlay results and endogenous deposition of SALSA pointed towards fibrinoids, the ECM, disrupted syncytiotrophoblast layers and necrotic villi as targets for SALSA. SALSA has previously been suggested to interact with fibrin/fibrinogen, platelets and erythrocytes [23]. Thus, we expected that SALSA could interact with components of the coagulation system or the ECM. First we tested whether SALSA has any effects on blood coagulation. Blood clotting was analyzed by measuring absorbance at 405 nm of citrated blood plasma after initiation of coagulation by BC Thrombin reagent (Siemens). Coagulation was compared between Maxisorp plate surfaces coated with or without SALSA. Fig 6 shows the clotting curve with averages and SD’s from 5 different measurements. On both surfaces the blood clot developed at the same speed and intensity as indicated by the increase in absorbance. Other basic coagulation assays such as Thrombin Time and Activated Prothrombin Time measurements were also performed with and without the addition of SALSA (0.5–5 μg/ml) into the plasma. Again, no effect of SALSA was observed (data not shown).

### Binding of SALSA to fibronectin

**Fig 7:**
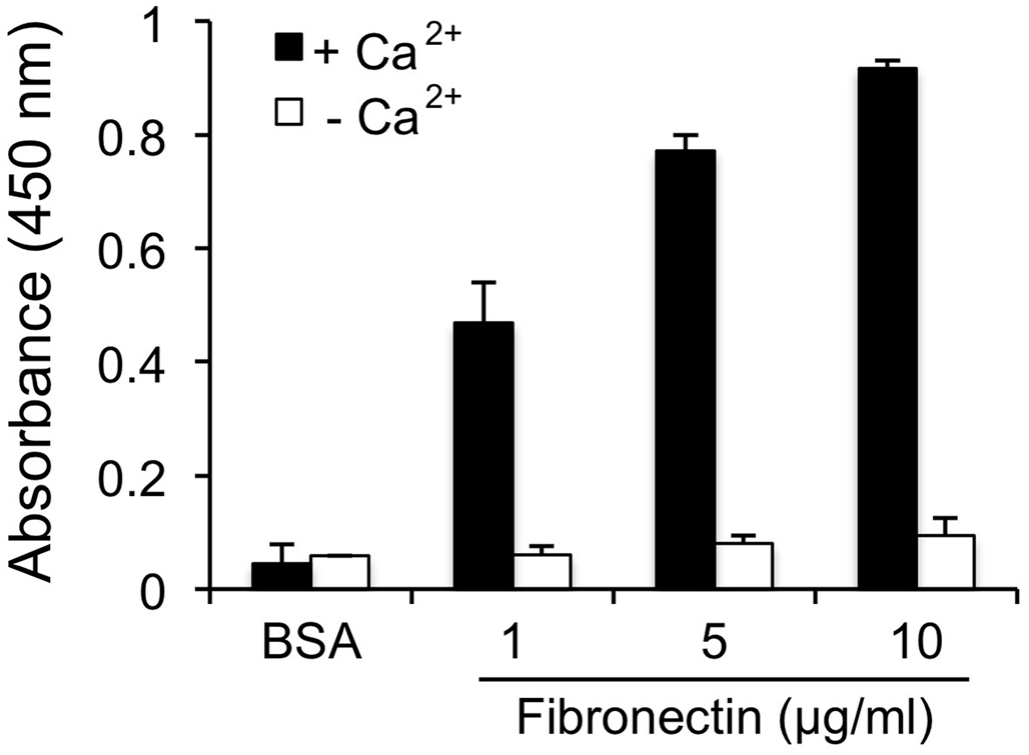
Binding of SALSA to the fibrinoid component fibronectin. Purified SALSA (1 μg/ml) was added to Maxisorp plate wells coated with fibronectin (1–10 μg/ml) and binding was monitored using monoclonal anti-SALSA antibodies. A clear calcium- and dose-dependent binding of SALSA to fibronectin was observed. Averages ± SD’s from three different experiments are shown.

**Fig 8:**
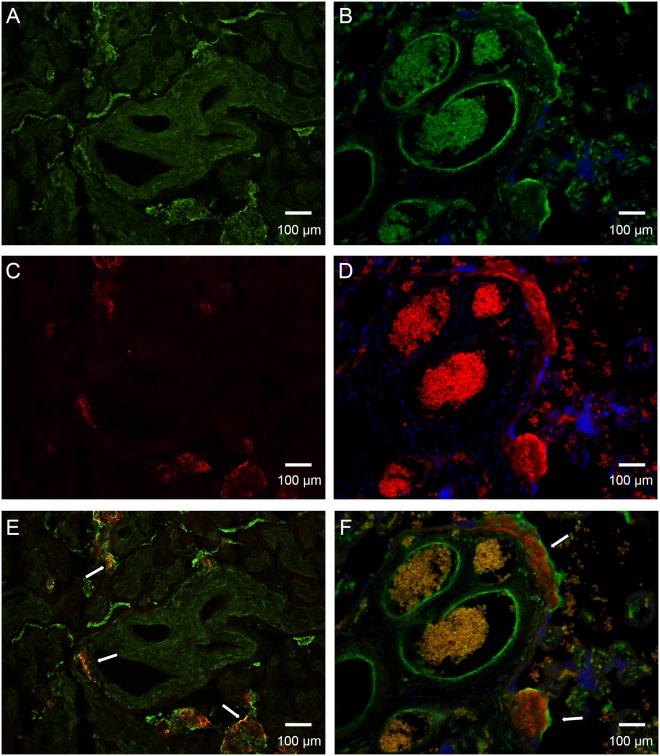
Comparison of SALSA co-localization with fibronectin and C1q. Placental sections of healthy term placentas were stained with a polyclonal anti-fibronectin antibody (A, green) and a polyclonal anti-C1q antibody (B, green). SALSA was stained using monoclonal anti-SALSA antibody (Hyb 213–06, panels C and D, red). Staining was visualized by immunofluorescence microscopy (200x magnifications). Superimpositions of the stainings are shown in (E) and (F). SALSA and fibronectin are partially found in the same structures in the placenta (yellow, white arrows in panel E). SALSA is located mainly in the inner mass of the fibrinoid structures, while fibronectin is mostly located at the edges of the structures. C1q and SALSA are also found in the same placental structures. However, no direct co-localization was observed. Instead, C1q appears to coat the SALSA-positive fibrinoids (white arrows in panel F). 200× magnifications.

To further analyze the targets of SALSA we next investigated binding of SALSA to fibrinoid components. As binding to fibrin has been shown previously [23], and we did not see an effect on blood coagulation, we focused on the major fibrinoid and ECM component, fibronectin. Fig 7 shows the results of an ELISA assay where fibronectin was coated in Maxisorp wells and purified SALSA was added. A clear calcium- and dose-dependent binding was observed between SALSA and fibronectin. Cellular fibronectin is not usually a component of fibrin-type fibrinoid, but plasma fibronectin is. We further studied whether SALSA co-localizes with fibronectin in placental tissues. In addition, based on the previous observations of C1q in fibrinoid structures [14], we also analyzed co-localization between SALSA and C1q. The proteins were visualized using specific antibodies on frozen (fibronectin) or paraffin-embedded (C1q) sections. A clear positive staining for both fibronectin and SALSA was observed (Fig 8, panels A, C and E). Interestingly, fibronectin and SALSA were found in the same fibrinoid structures in partial co-localization (Fig 8 panel E, yellow color, white arrows). Most SALSA staining was seen in the inner mass of the fibrinoid structures, while fibronectin was located at the edges of the structures. A similar type of staining pattern was observed for SALSA and C1q (Fig 8, panels B, D and F). When SALSA was seen deposited in necrotic villous structures or in fibrinoids, it appeared that C1q was located on the surfaces of these. C1q seemed to encapsulate the SALSA-positive areas. In most cases this had led to complement activation and C4 deposition (data not shown).

## Discussion

The presence of SALSA in AF and its ability to regulate complement prompted us to study SALSA in normal pregnancy and pregnancy complications. In this study we describe the presence of SALSA and its potential targets in AF and placenta. Increased SALSA levels were found in the first trimester AF samples from women who later developed PE. In early pregnancy SALSA was also found in the maternal decidual endothelium. In the placenta we found SALSA in the syncytiotrophoblast layer and in distinct extracellular formations called fibrinoids.

Specific findings in PE patients include maternal intravascular inflammation together with endothelial cell activation and/or dysfunction [3,5]. In the first trimester placentas, SALSA was present in decidua scattered in some capillaries, but not in others. The scattered staining suggests that the expression of SALSA is inducible under certain conditions in the endothelium from where it may deposit into the surrounding ECM. SALSA has not been detected in serum. However, SALSA may be induced in close proximity and then recognize structures in the injured endothelium or the surrounding ECM. In support of this, previous studies have demonstrated up-regulation of SALSA in epithelial cells in response to several inflammatory stimuli such as bacterial surface structures, tumor necrosis factor-α and interleukin 22 [35,36]. This is in line with observations made in the heart tissue, where SALSA was found to be inducible in the endothelium in pathological conditions, e.g. amyloidosis and bacterial endocarditis [23,37].

In this study we also found wide individual variation in SALSA levels in AF samples from healthy and complicated pregnancies. No significant differences in the relative levels of SALSA between the different disease groups were observed. In part, this may be due to the small sizes or various subpopulations of the sample groups. In comparison to healthy controls in early pregnancy, we did observe a trend of increased SALSA levels in AF from women who later developed PE. In both early and late PE samples two subpopulations with high or low SALSA levels were observed suggesting that PE patients may fall into distinct categories with regard to a possible involvement of SALSA in the pathogenetic process. Size polymorphisms of SALSA in AF have been described previously [27]. We tested if different variants would correlate with the pregnancy complications studied in Fig 1, but no correlation was found (data not shown).

Both the origin and the function of SALSA in AF are still uncertain. Our study revealed, for the first time, the presence of the SALSA protein in the placental tissue. Whether the SALSA protein observed in the AF and in the placenta both originate from the same source remains unclear. The results presented here indicate that SALSA is not produced by the uterine glands. Amniotic membranes could be one source for SALSA in the AF. However, our observations suggest that a possible constitutive expression there is very low (data not shown). Still, as expected for the decidual endothelium, the expression of SALSA could be inducible. Considering the abundant secretion of SALSA from mucosal membranes, the gastrointestinal and respiratory tracts of the fetus could also be sources of SALSA [27].

SALSA has been shown to interact with other pattern recognition molecules, including both C1q, MBL and and surfactant proteins A and D (SpA and SpD, respectively) [16,31,38]. In our earlier study we observed wide individual variation in the levels and the protein composition of SALSA in AF [27]. Several of the ligands of SALSA, including SpA, SpD and C1q interact with the collectin receptor, which has been found in the amniotic epithelium [39]. It is thus possible that SALSA in concert with these molecules affect signalling from fetal to maternal tissues. Within the uterus SALSA may have an increasingly important role during the maturation of the fetus towards parturition. From early pregnancy on (before 20 weeks of gestation) to term the relative levels of SALSA doubled from 0.15 ‰ to 0.3 ‰ of the total protein amount (*p*<0.05). This was matched by the observed stronger SALSA expression in term placentas. However, whether SALSA in the AF and placenta are functionally linked remains to be studied.

In term placentas immunohistochemical analysis showed SALSA in fibrin-type fibrinoid structures. This type of fibrinoid is mostly composed of fibrin together with other molecules from the coagulation system or from degenerative processes, including fibronectin [11]. The functions of fibrinoids have so far been linked to adapting the intervillous space to the altering flow conditions, to control the growth of the sprouting villous trees and to function as a substitute barrier wherever the continuity of the syncytiotrophoblast layer at the feto-maternal interface has broken down [11]. After performing several different tests to analyze the effect of SALSA on coagulation, we did not observe an affect on the formation of the fibrin clot itself. Instead, SALSA may be deposited after clot formation.

The lack of effect of SALSA on coagulation prompted us to search for other molecular targets in the fibrinoids. We found that SALSA bound to plasma-derived fibronectin in a dose- and calcium-dependent manner. This finding suggests that fibronectin could be a target for SALSA to get deposited into the fibrinoids. Fibronectin is also an ECM component, and thus could be a target for the SALSA deposition around endothelial cells as well, as was observed in the first trimester placentas. In term placentas we found fibronectin and SALSA in the same fibrinoid structures in the placenta with partial co-localization. *In vivo*, SALSA appears to interact with fibronectin directly. However, it is possible that other protein-protein interactions, such as binding to fibrin, are also involved in the fibrinoid structures.

SALSA has previously been implied in the activation of complement [29–31]. C1q and complement are known to function in the clearance of apoptotic cells and cellular debris [40]. Dysregulation of complement at the feto-maternal interface has for long been suspected to be part of the etiology of PE [41]. Indeed complement activation has in many cases been observed and recent studies found deposition of C4d on the syncytiotrophoblast layer in PE placentas [42]. No similar deposition was observed in healthy controls. In addition, our recent study described differences in deposition of C1q and the complement inhibitor C4b binding protein in fibrinoid structures between healthy and PE placentas [14]. SALSA has been shown to activate the classical and lectin pathways of complement, both leading to the deposition of C4b [29–31]. We therefore also investigated co-localization of SALSA and C1q. In this study, we found complement C1q deposited as a coating on the SALSA positive fibrinoids. Thus, SALSA deposition into fibrinoids may mediate complement targeting of the necrotic fibrinoid structures in the placenta.

SALSA was abundantly observed in areas, where the syncytial barrier between fetal and maternal tissue had broken down. SALSA was found in fibrinoids that either replaced destroyed syncytium, spread into the intervillous space or formed ring structures along the basement membranes of the syncytiotrophoblast. The ring formation likely interferes with the function of the villi by hindering the transfer of nutrients from the maternal to the fetal circulation. Previous studies have indicated that both SALSA and fibronectin are involved in epithelial differentiation [43–46]. When the syncytium is broken the formation of fibrinoid has been shown to be utilized by trophoblasts to re-epithelialize the villus [47]. SALSA and fibronectin could provide an ideal scaffold for this repair process in the developing placenta.

During the development of the placenta, and especially under pathological conditions, such as PE and other pregnancy complications, excessive placental blood flow irregularities and damage of the syncytium may occur. SALSA could then be induced locally at the same time as local fibrin-clot formation takes place. C1q has long been known for its housekeeping function, targeting and assisting phagocytosis of apoptotic cells and cellular debris [48]. We therefore suggest that in cases of local ischemia and tissue damage, the function of SALSA is to help contain the necrotic process and the excessive formation of fibrinoids through interactions with fibrin and fibronectin and thereafter participate in the targeting of C1q and complement to the tissue debris. In addition, the SALSA-positive fibrinoid formations may function as a scaffold for re-epithelialization of the villus.

## Abbreviations

AF: Amniotic fluid
DM: Diabetes mellitus type 1
DMBT1: Deleted in malignant brain tumors 1
ECM: Extracellular matrix
GDM: Gestational diabetes
IUGR: Intra-uterine growth restriction
MBL: Mannose binding lectin
PE: Pre-eclampsia
SAG: Salivary agglutinin
SALSA: Salivary scavenger and agglutinin
SpA: Surfactant protein A
SpD: Surfactant protein D
SRCR: Scavenger receptor cysteine-rich
